# Towards Robust Multiple Blind Source Localization Using Source Separation and Beamforming

**DOI:** 10.3390/s21020532

**Published:** 2021-01-13

**Authors:** Henglin Pu, Chao Cai, Menglan Hu, Tianping Deng, Rong Zheng, Jun Luo

**Affiliations:** 1 School of Electronic Information and Engineering, Huazhong University of Science and Technology, Wuhan 430074, China; puhenglin@hust.edu.cn (H.P.); dengtp@hust.edu.cn (T.D.); 2 School of Computer Science and Engineering, Nanyang Technological University, Singapore 639798, Singapore; chris.cai@ntu.edu.sg (C.C.); junluo@ntu.edu.sg (J.L.); 3 Department of Computing and Software, McMaster University, Hamilton, ON L8S 4L8, Canada; rzheng@mcmaster.ca

**Keywords:** microphone array layout, source separation, beamforming

## Abstract

Multiple blind sound source localization is the key technology for a myriad of applications such as robotic navigation and indoor localization. However, existing solutions can only locate a few sound sources simultaneously due to the limitation imposed by the number of microphones in an array. To this end, this paper proposes a novel multiple blind sound source localization algorithms using Source seParation and BeamForming (SPBF). Our algorithm overcomes the limitations of existing solutions and can locate more blind sources than the number of microphones in an array. Specifically, we propose a novel microphone layout, enabling salient multiple source separation while still preserving their arrival time information. After then, we perform source localization via beamforming using each demixed source. Such a design allows minimizing mutual interference from different sound sources, thereby enabling finer AoA estimation. To further enhance localization performance, we design a new spectral weighting function that can enhance the signal-to-noise-ratio, allowing a relatively narrow beam and thus finer angle of arrival estimation. Simulation experiments under typical indoor situations demonstrate a maximum of only $4^{\circ }$ even under up to 14 sources.

## 1. Introduction

Multiple blind sound source localization is an enabling technology for many practical applications such as indoor localization [1,2], radar sensing [3], and robotic navigation [4]. The typical underlying techniques for source localization is Angle of Arrival (AoA) estimation algorithms (hereafter, we will use AoA estimation to denote source localization as well) such as MUSIC [5,6], ESPRIT [7,8], SAGE [9], beamforming [10] or over-determined source separation and localization method [11]. Despite their success in obtaining correct AoA information even in the present of multiple sound sources, these common solutions have a important limitation: they can only locate a number of sources less than the number of microphones in an array. Meanwhile, these algorithms can only achieve sufficient resolution when the input sources have limited signal bandwidth. Otherwise, the performance would be significantly degraded. Using spectral weighting is a feasible approach to improve accuracy, which however, is vulnerable to interference [12].

Considering above limitations, another category of methods use binaural localization cues combined with Time-Frequency (T-F) masking for source separation which allows the separation of more number of input sources than the number of microphones. Motivated by the ability of 3D sound source localization via human ear, the authors of [13] present an algorithm for binaural localization with only two microphones. This algorithm process the mixture with Head Related Transfer Functions followed by a clustering technique, being able to locate multiple concurrent sound sources in both azimuth and elevation directions. However, this algorithm requires a prior knowledge on the number of input sources. In [14], the authors propose to use Interaural Time/Phase Differences (ILD and IPD) for separating and localizing multiple sound sources. However, the computational requirement for this algorithm is too high. A deep learning oriented mask based source separation algorithm is proposed in [15] while its performance is heavily dependent on the amount of training data, which makes the system relatively difficult to implement. In [16], a single channel based source separation algorithm is presented but only report satisfactory results at a limited number of input sources.

**Figure 1:**
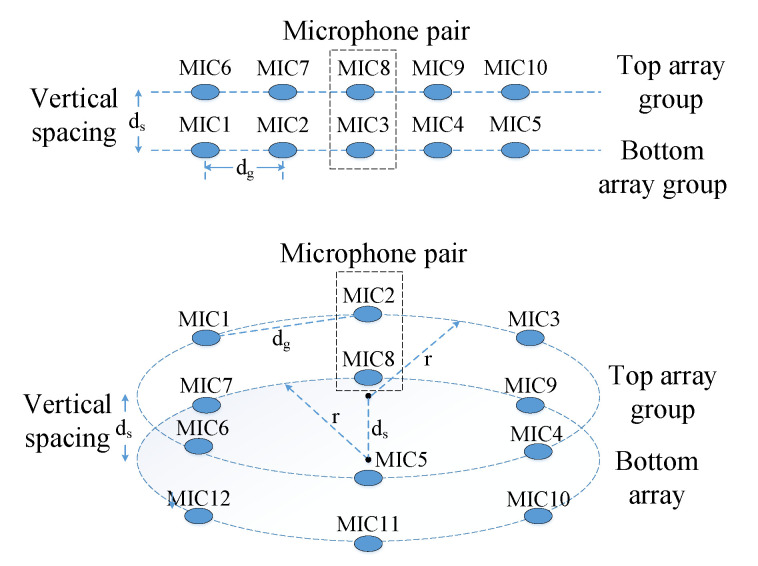
Microphone array structure. The upper linear array is appropriate for 2D source localization while the bottom two-layer circular array can be utilized in 3D scenarios.

To this end, in this paper, we propose a novel joint Source seParation and BeamForming algorithm (SPBF) for multiple blind source localization. Our algorithm is based on a novel microphone layout design shown in Figure 1. This layout design contains two groups of microphone arrays. One microphone array is on the top of another and the distance between these two arrays is kept small. The closest two microphones between two array groups make a pair and are used for source separation. Since the arrival time is preserved after source separation, we then individually exploit these de-separated signals for AoA estimation via delay-and-sum beamforming. Such separation-first-then-localization strategy allows to minimize mutual interference from different sound sources, therefore enabling finer AoA estimation. To further obtain accurate beamforming result, we introduce a new spectral weighting function, allowing to get a sharper beam pattern and thus not only provides better AoA estimation resolution but also higher SNR. The major contributions of this paper are summarized as follows:

- We design a new microphone array layout. With appropriate signal processing design, it allows to locate a number of sources larger than the number of microphones.
- We propose an new weighting function which can largely sharpen correlation peak hence finer AoA estimation resolution and higher SNR.

The rest of the paper is organized as follows. In Section 2, we describe our microphone array layout design consideration in details. Section 3 presents algorithm design. Simulation results are exhibited in Section 4. Finally, Section 5 concludes this paper.

## 2. Design of Microphone Layout

The structure of our proposed array layout is shown in Figure 1. It contains two groups of microphone arrays each has the same identical microphone layout. The closest distance between each microphone in an array group is denoted by $d_{g}$ and between two groups is $d_{s}$. The microphones in each array group are in the same plane. The two distances $d_{g}$ and $d_{s}$ should be properly configured so as to achieve the best performance.

The appropriate distance $d_{g}$ is determined by the bandwidth of input signals. More specifically, to avoid spatial aliasing, the following requirement should be satisfied [17]:

(1) $$d<\frac{\lambda_{\min}}{2},$$

where $\lambda_{\min}$ is the minimum wavelength for the signal of interest. Considering human hearing frequency range is 20 Hz∼20 kHz [18,19,20], the maximum distance between sensors should approximately be: $d_{\max}=\frac{\lambda_{\min}}{2}=\frac{c}{2f_{m}}\approx \frac{340}{2\times 20,000}\;m=0.085\;m$, where *c* is the sound speed, $f_{m}$ is the maximum frequency present in the sources. The above equation determines the upper bound for $d_{g}$.

The lower bound for $d_{g}$ is limited by beamforming algorithm. The accuracy of beamforming-based AoA estimation relies on the resolution of Time-Difference-of-Arrival (TDoA) among different microphones. This implies that $d_{g}$ should be as large as possible so as to maximize TDoA. Otherwise, high sampling rate would be needed, which however, requires computational resources and memory bandwidth, making it unsuitable for resource-constraints IoT devices. In particular, the observed sample sequences from two microphones that have the maximum distance in an array should have time delay no less than one sample. This implies that $d_{\min}=Kd_{g}>t_{\delta }=\frac{c}{f_{s,\max}}$, where $t_{\delta }$ denotes the time delay incurred by one sample and $f_{s,\max}$ denotes the maximum sampling rate. For instance, on a circular array with 6 microphones working at 16 kHz, $d_{\min}=2d_{g}>t_{\delta }=\frac{c}{f_{s,\max}}=\frac{340}{16,000}\approx 2$ cm, so $d_{g}>1$ cm. This constraint can also help to obtain the maximum operational sampling rate for a given array.

The distance $d_{s}$ affects the performance for source separation. In our design, we utilize Degenerate Unmixing Estimation Technique (DUET) algorithm that only requires two microphones whose distance should be as small as possible so as to prevent phase-warping. To this end, $d_{s}$ should be minimized to its physical limitation. Under this case, the source separation algorithm can achieve optimal performance and preserve as much spatial information for each source as possible.

## 3. Robust Source Localization

Our robust source localization involves two steps: blind source separation and beamforming based localization.

### 3.1. Blind Source Separation

To separate mixed signals, we utilize Degenerate Unmixing Estimation Technique (DUET) algorithm [21]. DUET is able to blindly separate an arbitrary number of sources given just two anechoic mixtures namely, two microphones, provided that the time– frequency representations of sources are *disjoint* [21,22] or non-overlapped entirely, which is true under most cases [21,23]. The demixing processing can thereby be deemed as a partitioning in time-frequency plane.

In DUET, the received mixed signals by two microphones in the time-frequency domain can be written in a simple form as:

(2) $$\left[\begin{array}{c} {\hat{x}}_{1}\left(\tau ,\omega \right)\\ {\hat{x}}_{2}\left(\tau ,\omega \right) \end{array}\right]=\left[\begin{array}{c} 1\\ a_{j}e^{-i\omega \delta_{j}} \end{array}\right]{\hat{s}}_{j}\left(\tau ,\omega \right)\;for\;some\;j.$$

where ${\hat{x}}_{1}\left(\tau ,\omega \right)$ denotes the time-frequency representation of the first microphone, ${\hat{s}}_{j}\left(\tau ,\omega \right)$ is original source signals without delay and attenuation. In the above equation, the subscript *j*, known as the active index *j*, indicates which source dominates current frame. The core principle behind DUET is that the ratio of the time–frequency representations can fully characterize the mixing parameters:

(3) $$\forall \left(\tau ,\omega \right)\in \Omega_{j},\;\frac{{\hat{x}}_{2}\left(\tau ,\omega \right)}{{\hat{x}}_{1}\left(\tau ,\omega \right)}=a_{j}e^{-i\omega \delta_{j}}$$

where $\Omega_{j}:=\left\{\left(\tau ,\omega \right):{\hat{s}}_{j}\left(\tau ,\omega \right)\neq 0\right\}$. The mixing parameters, namely the local attenuation estimator $\tilde{a}\left(\tau ,\omega \right)$ and the local delay estimator $\tilde{\delta }\left(\tau ,\omega \right)$, can be calculated based on the active source component:

(4) $$\tilde{a}\left(\tau ,\omega \right):=|x_{2}\left(\hat{\tau },\omega \right)/x_{1}\left(\hat{\tau },\omega \right)|,$$

(5) $$\tilde{\delta }\left(\tau ,\omega \right):=\left(-1/\omega \right)∠\left(x_{2}\left(\hat{\tau },\omega \right)/x_{1}\left(\hat{\tau },\omega \right)\right)$$

The above equation helps to label the current active source. Therefore, in the following steps, we can demix the mixture via binary masking constructed on these mixing parameters. To accomplish this task, for each active index *j*, an indicator functions is built:

(6) $$M_{j}\left(\tau ,\omega \right)=\left\{\begin{array}{cc} \; & 1\;\left(\left(\tilde{a}\left(\tau ,\omega \right)\right),\left(\tilde{\delta }\left(\tau ,\omega \right)\right)\right)=\left(a_{j},\delta_{j}\right)\\ \; & 0\;otherwise \end{array}\right.$$

At this step, we can demix the sources now:

(7) $${\tilde{\hat{s}}}_{j}\left(\tau ,\omega \right)={\tilde{M}}_{j}\left(\tau ,\omega \right)\left(\frac{{\hat{x}}_{1}\left(\tau ,\omega \right)+{\tilde{a}}_{j}e^{i{\tilde{\delta }}_{j}\omega }{\hat{x}}_{2}\left(\tau ,\omega \right))}{1+{\tilde{a}}_{j}^{2}}\right)$$

To further enhance the performance, clustering techniques are applied on the estimated attenuation and delay parameters. The number of clusters is the estimated number of sources and the cluster centres are often deemed as the optimal estimation of the mixing parameters for each source. At the last step, we can reconstruct the sources from the time–frequency representations by converting back into the time domain.

### 3.2. Beamforming and Localization

After demixing multiple sources, we utilize beamforming method to locate their angle-of-arrival.

#### 3.2.1. Beamforming Process

The delay-and-sum (hereafter, we will call it vanilla) beamforming [17,24] finds the incident angle by searching the maximal energy over a spherical grid when using a circular array (hereafter, we assume we adopt a circular array). Supposing there are M-microphones, a beamformer output can be defined as:

(8) $$y\left(n\right)=\sum_{m=1}^{m=M}x_{m}\left(n-\tau_{m}\right),$$

where $x_{m}\left(n\right)$ is the signal from the *m*th microphone and $\tau_{m}$ is the respective arrival time delay. The energy of beamformer output over a frame window of *N* is thus formulated as:

(9) $$e=\sum_{n=1}^{n=N}y{\left(n\right)}^{2}.$$

Using Equations (8) and (9) can be expanded as:

(10) $$\begin{array}{c} e=\sum_{m=1}^{m=M}\sum_{n=1}^{n=N}x_{m}^{2}\left(n-\tau_{m}\right)\\ +2\sum_{m_{1}=1}^{m_{1}=M}\sum_{m_{2}=2}^{m_{2}=M}\sum_{n=1}^{n=N}x_{m_{1}}\left(n-\tau_{m_{1}}\right)x_{m_{2}}\left(n-\tau_{m_{2}}\right)\\ =C+2\sum_{m_{1}=1}^{m_{1}=M}\sum_{m_{2}=2}^{m_{2}=M}R_{m_{1}m_{2}}\left(\tau_{m_{1}}-\tau_{m_{2}}\right), \end{array}$$

where the term $\sum_{m=1}^{m=M}\sum_{n=1}^{n=N}x_{m}^{2}\left(n-\tau_{m}\right)$ can be regarded as a constant since τ is relatively small, and $R_{m_{1}m_{2}}$ denotes cross-correlation between microphone $m_{1}$ and $m_{2}$. Since τ can be parameterized by incident angle θ, the problem of AoA estimation via beamforming can thus be formulated as:

(11) $$\phi =\underset{\theta }{\arg\max}e\left(\tau |\theta \right).$$

where ϕ is the estimated optimal incident angle.

The afore-mentioned optimization problem is often solved by a greedy search that often leads to excesive computation power. To reduce computational energy, the solution space is often discretized and a lookup table between θ and τ is constructed so as to efficiently obtain e(τ|θ). The relation between θ and τ is calculated based on far-field assumption:

(12) $$\tau_{ij}=\frac{f_{s}}{c}\left({\vec{d}}_{i}-{\vec{d}}_{j}\right)\vec{u},$$

where ${\vec{d}}_{i}$ and ${\vec{d}}_{j}$ are the positions of the *i*th and *j*th microphones, respectively, $\vec{u}$ is the unit vector indicating the direction of a point source, $f_{s}$ is the sampling rate, and *c* is the speed of sound in air.

#### 3.2.2. Problem of Vanilla Beamforming Algorithm

The performance of vanilla beamforming algorithm largely depends on cross-correlation. If an incident source has good compression properties [25] where its correlation peak is sharp, the algorithm would get narrow beam width hence better estimation resolution and high SNR. However, in most cases, the sources do not hold such properties, making the beam rather wide hence subject to background interference.

#### 3.2.3. Spectral Weighting Function

Spectral weighting are common approaches to sharpen the beam width, among which GCC-PHAT [26] is the most popular one. However, in GCC-PHAT, each frequency bin of the spectrum contributes equally to the final correlation, making it sensitive to interference.

To balances the contribution of each frequency bin to correlation results, we propose a new weighting function.

(13) $$W\left(\omega \right)=\frac{G(\omega )}{{\left|X\left(\omega \right)\right|}^{\rho }},$$

where $\rho =\max\{\beta ,\frac{X\left(\omega \right)-\alpha X_{\sigma }\left(\omega \right)}{X(\omega )}\}$, $X_{\sigma }\left(\omega \right)$ is the mean spectral power of noise, estimated in the absence of source signals, α≤1 is a coefficient quantify how conservative the estimated noise power is (default to 0.9), β is normally set to 0.4. G(ω) is Wiener function of *prior* SNR ξ, given by:

(14) $$G^{n}\left(\omega \right)=\frac{\xi^{n}}{\xi^{n}+1},$$

where $\xi^{n}=\frac{E\{{\left[X^{n}\left(\omega \right)\right]}^{2}\}}{E\{{\left[X_{\sigma }^{n}\left(\omega \right)\right]}^{2}\}}$, and the super script *n* here refers to *n*th time frame. The numerator $E\{{\left[X^{n}\left(\omega \right)\right]}^{2}\}$ in $\xi^{n}$ could be estimated using decision-directed approach [27]:

$${\hat{\xi }}_{n}=\frac{\gamma {\left[G^{n-1}\left(w\right)\right]}^{2}{\left|X^{n-1}\left(w\right)\right|}^{2}+\left(1-\gamma \right){\left[X^{n}\left(w\right)\right]}^{2}}{E\left\{{\left[X_{\sigma }^{n}\left(w\right)\right]}^{2}\right\}},$$

where γ is a constant and normally is set to 0.9.

## 4. Simulation

We conduct extensive simulations to evaluate the performance of our proposed algorithm.

### 4.1. Setup

**Figure 2:**
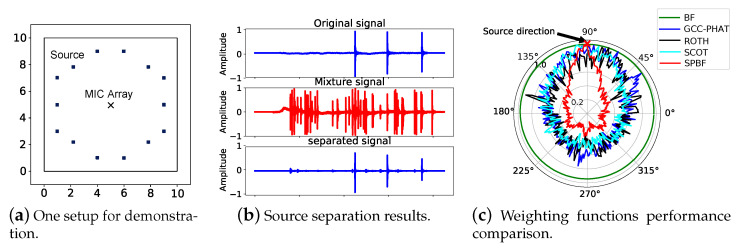
Setup and results. (**a**) shows the setup that has up to 14 different sound sources around our designed microphone array (12 mics) and these sources with a duration of 20∼50 ms are triggered simulatenously. (**b**) demonstrates that DUET is able to distangle multiple sources. (**c**) depicts that our proposed weighting function achieve better performance than other algorithms when there is only one input source at a relatively low SNR.

We evaluate the performance in a typical indoor room with a size of 10 × 10 × 3 m ${}^{3}$ and simulate the multipath signals via ray tracing technique as shown in Figure 2a. Figure 2a depicts a simple setup for demonstration where fourteen types of real-recorded footstep sounds are emitted from different locations as source signals and we place our microphone array in the center of the room. The signal duration is between 20∼50 ms and they are triggered simultaneously. This microphone array contains two cascaded circular sub-arrays as shown in Figure 1. The microphone distance in each group is configured as 4.5 cm and for each pair is 1 cm. Therefore, our array has a total of twelve microphones.

### 4.2. Results

We first demonstrate the performance of each component in SPBF. To start with, we show that DUET is feasible to decompose multiple sound sources with only two microphones and the results are shown in Figure 2b. The results reveal that the demixed signal is highly consistent with the original one. We then inspect the impacts of our proposed weighting function on the beamforming algorithm. In this experiment, we utilize only one sound source (footstep sounds). For comparison, we also implement GCC-PHAT [28] along with several other existing work including ROTH [29] and Smoothed coherence transform(SCOT) [30], the results are shown in Figure 2c. It is observable that our algorithm cannot only locate the ground truth AoA but also exhibit a rather narrow beam pattern, making it noise-resilient. While in comparison, GCC-PHAT, ROTH, and SCOT have many noisy peaks, making it infeasible to correctly locate even a single sound source. Vanilla beamforming algorithm though can identify the correct AoA but have rather wide beam patterns, making it vulnerable to interference. The results clearly demonstrate the effectiveness of our weighting function.

**Figure 3:**
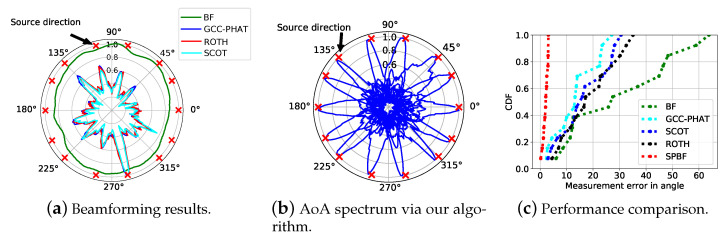
(**a**) shows the AoA spectrum between different algorithms. (**b**) demonstrates the AoA spectrum using our first separation and then beamforming algorithm, yet with proposed spectral weighting function. (**c**) Source localization performance between different algorithms.

**Figure 4:**
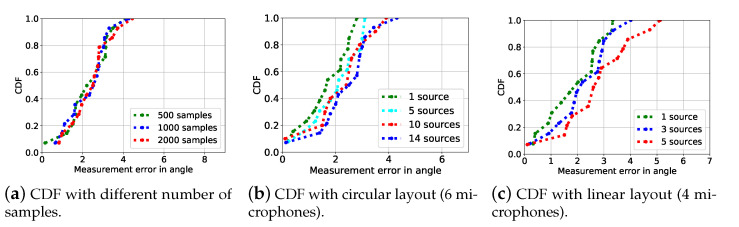
(**a**) depicts the results of source localization under different number of samples, which shows no obvious differences. Source localization errors under different number of sources with circular array (**b**) and linear array (**c**)

We next check the beamforming results with common algorithms including GCC-PHAT, ROTH, and SCOT in Figure 3a and our SPBF in Figure 3b. It is obersevable that AoA spectrum of SPBF is sharper and has less side lobes than other algorithms, indicating the effectiveness of our proposed algorithm. We have also extensively evaluate the localization performance between vanilla beamforming, GCC-PHAT, SCOT, ROTH, and SPBF. The results presented in Figure 3c demonstrates that the 80-percentile error of SPBF is at least 5.5× improvements over other algorithms. The results clearly demonstrate the superior performance of our proposed algorithm. In the following experiment, we explore the impact of the number of samples on the localization performance and the results are shown in Figure 4a. It is observable that the number of samples or equivalently the duration of signals impose negligible impacts on the localization performance. Since our algorithm can effectively work at a minimal of 500 samples (equivalently 10 ms given a sampling rate of 48 kHz), it is robust to the Doppler effect. We then conduct experiments when the number of sources is larger than the number of microphones and the results are shown in Figure 4b. The results reveal that the number of sources does not impose significant impacts on beamforming results. SPBF can achieve a median accuracy of $2.5^{\circ }$ and a 80-percentile of $1.7^{\circ }$. The maximum error does not exceed $5^{\circ }$. We also conduct experiments using a linear array. The results in Figure 4c reveal that the performance of circular array is slightly better than that of linear array due to more microphones.

**Figure 5:**
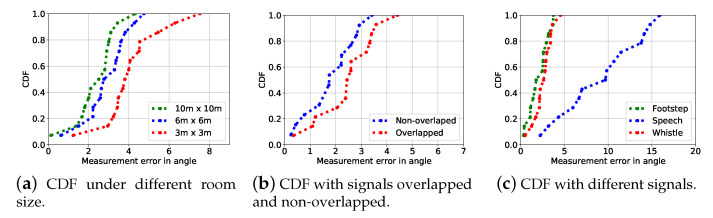
Source localization errors under different room size (**a**), emulating the severity of multipath effect. (**b**) The source localization errors with signals overlapped and non-overlapped exhibit no obvious differences. (**c**) Source localization errors with different type of signals.

We explore the impact of multipath effect by operating the source localization algorithms in different room size and the results are shown in Figure 5a. It is observable that the multipath effect can affect the source localization performance as the localization errors increase when the room size becomes smaller, equivalent more severe multipath effect. We next explore the results when multiple sources transmit non-overlapped (non-concurrently) and overlapped (concurrently). The results shown in Figure 5b reveal that when the sources are overlapped, the performance experience no obvious performance drop, indicating the robustness of our algorithm. We finally perform localization using different types of souce signals including footstep, speech, and whistle and the results are shown in Figure 5c. It can be observed that different types of source inputs can have a large impact on the final performance. As we can see from Figure 5c, the localization performance of footstep and whistle are significantly better than speech. This is due to the fact that footstep and whistle have much more sharp auto-correlation peaks than speech, making the final localization performance much better.

### 4.3. Runtime Performance

We have explored the time cost of each module of our proposed algorithm and report the results in Table 1. The results are obtained on Intel(R) Core(TM) i7-6700HQ CPU @2.60 GHz with 8 GB RAM. The code is implemented in C language. The time overhead for each module in Table 1 is the average result of 100 trials. The source separation module that consumes 262.59 ms dominates the overhead of the proposed algorithm. The time cost for spectral weighting and beamforming is 11.68 ms and 95.76 ms, respectively. Therefore, the total time overhead is 370 ms, which achieves satisfactory runtime performance. We believe that when involving more advanced code optimization technique or hardware architecture such as GPU, the runtime performance can be further improved.

## 5. Conclusions

In this paper, we propose to first separate mixed sources and then utilize beamforming with proposed spectral weighting function to locate multiple sources. In particular, we design a new microphone layout that enables top locate more number of sources than the number of microphones in an array. Simulation results demonstrate that the proposed algorithm can achieve significantly better performance than existing solutions.

## Figures and Tables

**Table 1:** Computational time cost in ms for each module.

Module	Source Separation	Weighting	Beamforming
Time cost (ms)	262.59	11.68	95.76

## Data Availability

Data available on request due to privacy restrictions.

## Abbreviations

The following abbreviations are used in this manuscript:

SPBF	Source seParation and BeamForming
AoA	Angle of Arrival
MUSIC	MUltiple SIgnal Classification
ESPRIT	Estimation of Signal Parameters via Rational Invariance Techniques
SAGE	Space-alternating Generalized Expectation-maximization
SNR	Signal-to-noise Ratio
TDoA	Time-Difference-of-Arrival
DUET	Degenerate Unmixing Estimation Technique
GCC-PHAT	Generalized Cross-correlation with Phase Transform
SCOT	Smoothed coherence transform
